# Enhanced Degradation of Rh 6G by Zero Valent Iron Loaded on Two Typical Clay Minerals With Different Structures Under Microwave Irradiation

**DOI:** 10.3389/fchem.2018.00463

**Published:** 2018-10-09

**Authors:** Wenxiu Rao, Guocheng Lv, Danyu Wang, Libing Liao

**Affiliations:** Beijing Key Laboratory of Materials Utilization of Nonmetallic Minerals and Solid Wastes, National Laboratory of Mineral Materials, School of Materials Science and Technology, China University of Geosciences, Beijing, China

**Keywords:** nanoscale zero valent iron, kaolinite, sepiolite, microwave, Fenton-like reaction

## Abstract

Nanoscale zero valent iron has been a widespread concern in various fields due to its large specific surface area and high reactivity. However, nanoscale zero valent iron (nZVI) is very likely to aggregate and be oxidized, which limit its wide application in industry. Most clay minerals have a large adsorption capacity of cations due to their negative charges and high specific surface areas. In the present work, nZVI was loaded onto two typical clay minerals: kaolinite and sepiolite, to inhibit its oxidation and aggregation. The composites were applied to degrade Rhodamine 6G (Rh 6G) under microwave irradiation. The effects of pH value and microwave power on degradation were studied. The results showed that the removal amount of Rh 6G by nZVI/kaolinite was 110 mg/g in 15 min, while it reached 300 mg/g by nZVI/sepiolite. The difference between these two composites was mostly determined by the structures of these two clay minerals.

## Introduction

About 80% of the textile wastewater comes from the textile printing and dyeing industry every year, which is an industrial sector with large water consumption and high discharge of wastewater (Liu et al., 2017). Printing and dyeing wastewaters have the characteristics of large water volume, high chroma and high pollutant concentration (Zhao et al., 2010). Textile processing wastewater contains a variety of contaminants. Direct discharge of the wastewater without treatment into rivers and lakes would cause serious pollution to environment and bring a serious threat to the survival of mankind (Kehinde and Aziz, 2016). However, the treatment of wastewater is extremely difficult, which made it necessary to develop new materials to process the wastewater (Liu et al., 2015).

Clay minerals are mainly composed of silicon, aluminum and magnesium. They are inexpensive and abundant in nature all over the world, and play an important role in environmental protection and the development of new mineral materials (Kelm et al., 2003). Clay minerals have complex pore structures and high specific surface areas (Steudel et al., 2009). We want to load nZVI on clay minearls' surface to make more sufficient contact with the solution. Kaolinite is a typical 1:1 layered structure, its lattice structure is very orderly, so there is basically no isomorphous substitution in its structure, resulting in poor swelling performance in water (Ezzatahmadi et al., 2017), sepiolite also can't spontaneously swell in water, therefore nZVI can only be supported on their surface. Kaolinite can be used to adsorb many organic pollutants, due to its special layered structure, such as congo red dye (Vimonses et al., 2009), heavy metals (Matłok et al., 2015) and dipalmitoyl lecithin (Jr et al., 1975). Sepiolite is a common 2:1 chain layered type of clay mineral, composed of two layers of silicone tetrahedron and an intermediate layer of magnesium octahedron (Kaviratna, 1994). It can also adsorb numerous organic pollutants, such as reactive blue 221 (Alkan et al., 2007), 3-aminopropyltriethoxysilane (Demirbaş et al., 2007) and β-carotene (Sabah et al., 2007).

Fenton reaction of degradation of organic dyes attracted considerable attention in recent years (Cheng et al., 2008), using the catalytic power of Fe^2+^ to generate highly reactive hydroxyl radicals (HO^•^) to degrade organic dyes (Masomboon et al., 2010). However, Fenton reaction requires high concentrations of iron ions (Yoon et al., 2001) and an acidic reaction environment (Liu et al., 2013), greatly increasing the cost of degradation of organic dyes. Fenton-like reaction can degrade organic dyes using nZVI (Xu and Wang, 2013), which can solve the problems caused by Fenton reaction mentioned above. nZVI can degrade many organic pollutants due to its special properties, such as 2,4-dichlorophenol (Li et al., 2018), trichloroethylene (TCE) (Kim et al., 2010), methylene blue (Yang et al., 2015). However, due to the aggregation and oxidation of nZVI (Dong et al., 2017), the loading of nZVI onto clay minerals is expected to inhibit the aggregation and oxidation of nZVI.

It has been proven that microwave could accelerate the rate of catalytic reactions (Mochizuki et al., 2015; Bianchi et al., 2017). The aqueous solutions in the reaction systems can self-heat by absorbing microwaves (Zhang and Liao, 2017), and the reaction rate can be greatly improved.

In the present work, nZVI was loaded onto kaolinite and sepiolite, respectively, and used to degrade Rh 6G under microwave irradiation. It was found that the removal amount of Rh 6G by nZVI/sepiolite was greater than that of nZVI/kaolinite. The loading amount of nZVI on sepiolite was almost three times as that on kaolinite, which was mainly determined by the structures of the clay minerals (Graphical Abstract).

**Graphical Abstract:**
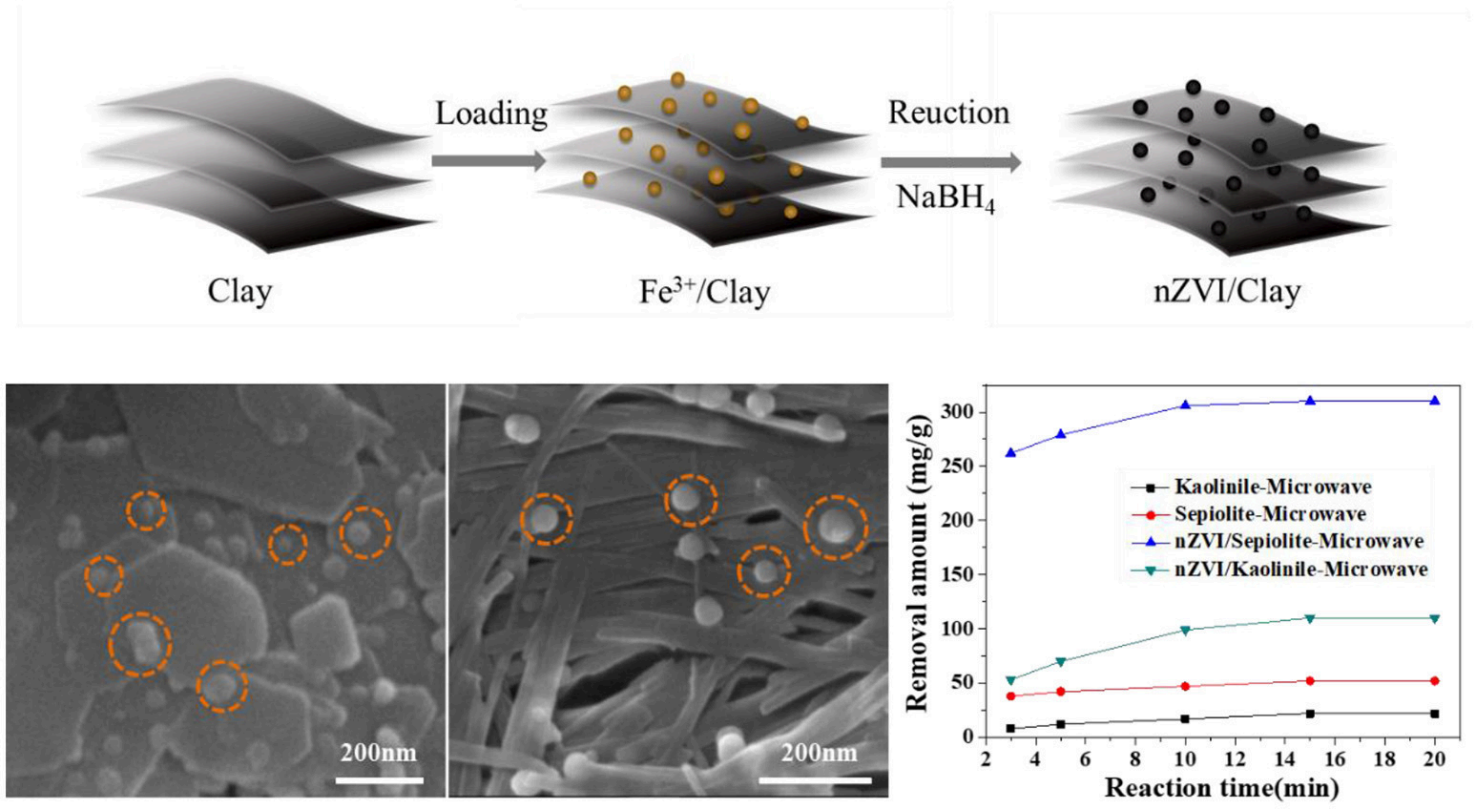
nZVI/Clay's synthesis process, surface topography and degradation performance of Rh 6G.

## Experimental

### Materials

Iron (III) chloride hexahydrate (FeCl_3_·6H_2_O) and sodium borohydride (NaBH_4_) were purchased from Beijing Chemical Workstation. Rh 6G was from Aladdin. All the chemicals were of analytical grade. The sepiolite was obtained from Sigma Aldrich, its BET area and average pore size are 319.4 m^2^/g and 6.885 nm, respectively. The kaolinite was bought from China Kaolin Co, Ltd, its BET area and average pore size are 22 m^2^/g and 30.095 nm, respectively. All the chemicals and minerals were used without further purification. All of the solutions were formulated with distilled water.

### Synthesis of nZVI/kaolinite and nZVI/sepiolite

100 mL distilled water, 5 g kaolinite (or sepiolite) and 10 g FeCl_3_·6H_2_O were added in a three-necked flask and stirred for 12 h to load Fe^3+^ on clay minerals. Then 8 g NaBH_4_ was added, and stirred for another 30 min under the protection of nitrogen at room temperature. The suspension was centrifuged and washed with ethanol for three times. The solid part was then freeze-dried.

### Rh 6G removal experiment

2,000 mg Rh 6G was dissolved in 500 mL distilled water and ultrasonic for 15 min to prepare 4,000 mg/L Rh 6G solution. In batch experiments, 0.1 g as prepared sample was added in 10 mL Rh 6G solution, then, the mixture was placed in a microwave oven to degrade Rh 6G for a certain time under different microwave power. After the degradation, the mixture was centrifuged and the supernatant was filtered through 0.22 μm syringe filters before being taken to a UV-Vis spectrophotometer for the analysis of equilibrium concentrations. In the cycle test, the used nZVI/clay was reduced by NaBH_4_ before the next trial. All experiments were run in duplicates.

### Characterizations

The crystal diffraction data was determined by the X-ray diffractometer (Rigaku D/Max-IIIa X-ray diffractionmeter) with a CuKα-radiation at 30 kV and 20 mA operating conditions. Angle ranged from 3 to 70° and the speed was 8° min^−1^ with a scanning step length of 0.01°. Scanning electron microscopy (SEM) was used to record the surface morphology of the samples, carried out on a scanning electron microscope (JSM-IT300) with conditions at 30 kV and 10^−4^ Pa. The equilibrium concentration of Rh 6G was determined by a UV-Vis spectrophotometer (UV2400/PC), the value of the absorption coefficient at 525 nm. The surface compositions of samples were tested by an X-ray photoelectron spectrometer (K-Alpha), which can analyze the valence of elements in samples, especially valence state of iron element. The microwave network analyzer N5244A (Agilent) was used to analyze microwave absorption properties of flexible absorbing film. The frequency range was from 2 to 10 GHz. Automated gas sorption analyzer (Autosorb-iQ-2MP) was used to analyze the specific surface area of the samples. Zeta potential was recorded by nanoparticle size potential analyzer (Zetasizer Nano ZS90). The coaxial wire method was adopted for the analysis.

## Results and discussion

**Figure 1:**
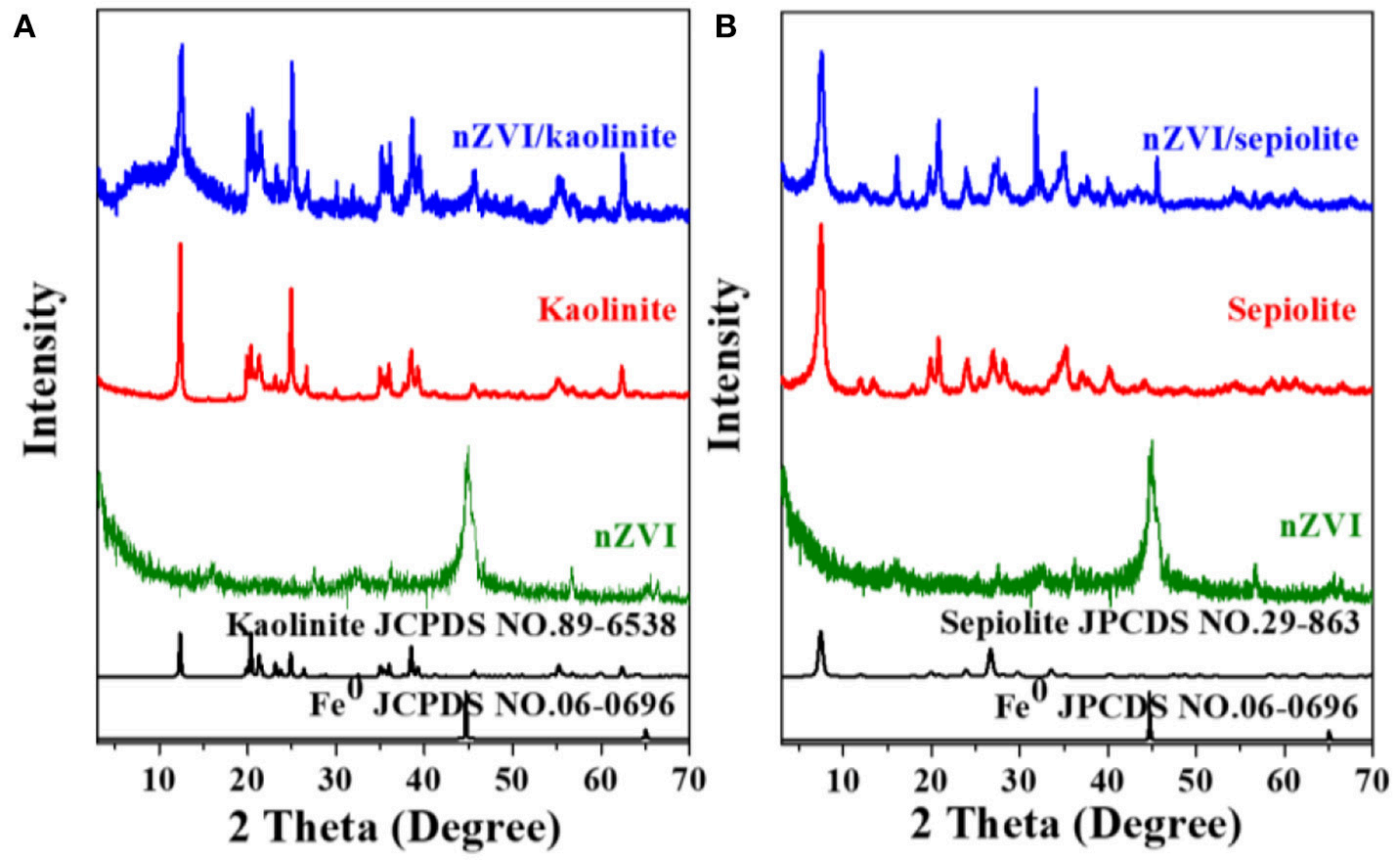
X-ray diffraction patterns. Kaolinite, nZVI, and nZVI/kaolinite **(A)**; Sepiolite, nZVI, and nZVI/sepiolite **(B)**.

The composites were synthesized by liquid phase reducing method. The structures of nZVI, kaolinite, speioilte and the composites were showed in Figure 1. The reflections at 2θ = 12.38° (Figure 1A), 7.58° (Figure 1B) corresponded to the (001) basal plane of kaolinite (JCPDS No. 89-6538) and the (001) basal plane of sepiolite (JCPDS No. 29-863), respectively. The diffraction peak at 44.7° is attributed to the (110) basal plane of Fe^0^ (JCPDS No. 06-0696). No obvious characteristic peaks of iron oxides was founded in the nZVI/kaolinite or nZVI/sepiolite, indicating that nZVI was not oxidized or only a very small part was oxidized during the synthesis process. Besides, the patterns of the composites stayed almost the same before and after the loading, suggesting that the structures of clay minerals not yet destroyed, and nZVI is loaded on the surface of mineral material rather than intercalated in this study.

**Figure 2:**
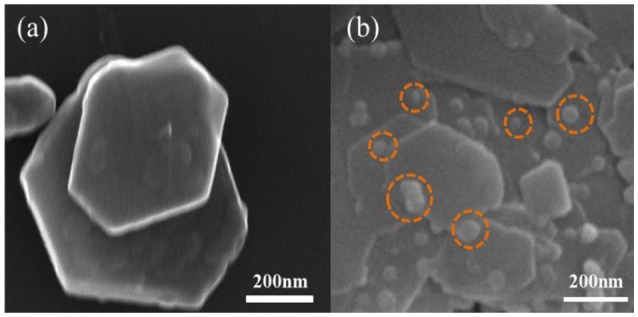
SEM image of kaolinite **(a)**, nZVI/kaolinite **(b)**.

**Figure 3:**
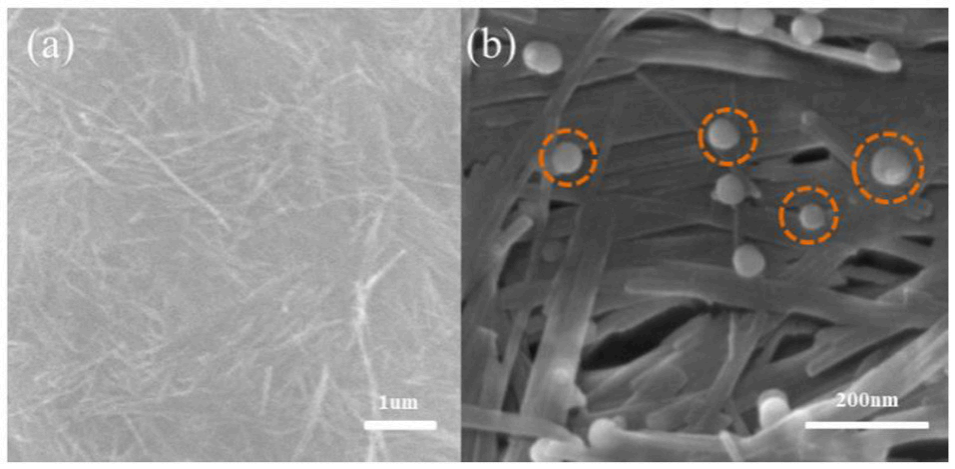
SEM image of sepiolite **(a)**, nZVI/sepiolite **(b)**.

The SEM results in Figures 2, 3 revealed the morphology of the minerals before and after the loading of nZVI. The lamellar structure of kaolinite was observed clearly. nZVI particles were evenly distributed on the layers of kaolinite, within a size range of 25–35 nm, indicating that the loading of nZVI on kaolinite could suppress the aggregation of nZVI effectively.

While loading onto sepiolite, most of the nZVI particles were attached to the fibers within a size range of 20–45 nm.

**Figure 4:**
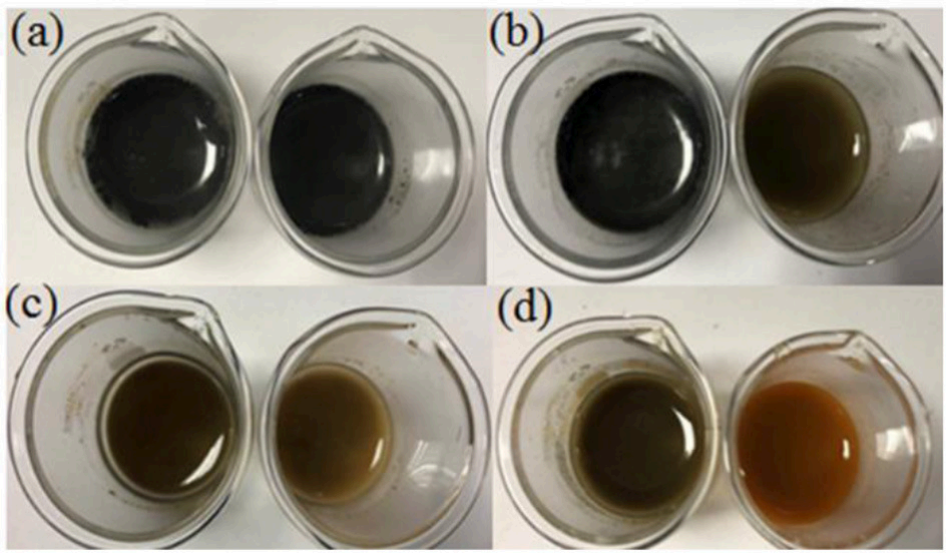
Color changes of nZVI, nZVI/clay in water after a certain time: 0 h **(a)**, 12 h **(b)**, 36 h **(c)**, and 72 h **(d)**.

Figure 4 showed the color changes of nZVI and nZVI/clay composites after dispersed in water for different amounts of time.

The color of materials changed to black after the addition of nZVI and nZVI/clay. As time went by, the color of both materials become lighter gradually due to the oxidation of nZVI. However, nZVI/clay showed a better resistance to oxidation than nZVI as the color of nZVI suspension changed from black to yellow completely after 72 h while the color of nZVI/clay suspension just started to turn yellow after the same time. It indicated that only a small portion of nZVI was oxidized after loading, which means that loading nZVI onto clay could effectively inhibit its oxidation.

**Figure 5:**
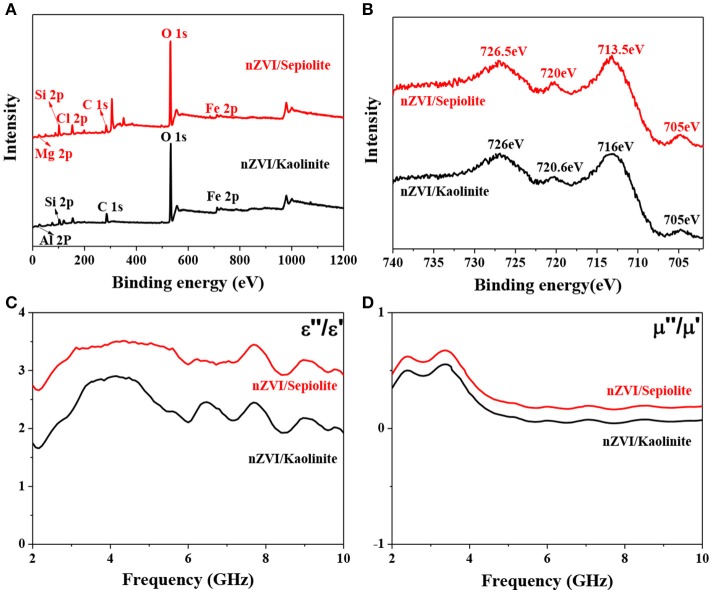
XPS survey spectrum of nZVI/kaolinite and nZVI/sepiolite **(A)**. XPS fine spectrum of Fe 2p **(B)**. Frequency dependence of nZVI/kaolinite and nZVI/sepiolite of the dielectric loss **(C)** and magnetic loss **(D)**.

The surface compositions of nZVI/kaolinite and nZVI/sepiolite and the valence state of Fe on its surface were analyzed by XPS technique. The survey spectrum of this sample was shown in Figure 5A, proving that the composites contains iron elements. The fine spectrum of Fe 2p of nZVI/kaolinite and nZVI/sepiolite contains four different peaks, as shown in Figure 5B. The peaks at binding energies of 705 and 720 eV could be assigned to the 2p3/2 and 2p1/2 peaks of Fe^0^ in nZVI/sepiolite and nZVI/kaolinite. The other two peaks at binding energies of 713.5 and 726.5 eV could be attributed to the 2p3/2 and 2p1/2 peaks of Fe (III) in nZVI/sepiolite while 716 and 726 eV in nZVI/kaolinite, indicating that a small part of Fe^0^ on the surface of the composite was oxidized to Fe (III).

The ability of a substance to absorb microwaves is mainly determined by its dielectric loss factor. A substance with a large dielectric loss factor usually has a strong ability to absorb microwaves. On the contrary, a material with a small dielectric loss factor has a weak ability to absorb microwaves. The dielectric loss (tan δe = ε″/ε′) of nZVI/kaolinite and nZVI/sepiolite was analyzed by a microwave network analyzer (Figure 5C). The dielectric loss of nZVI/sepiolite was higher than that of nZVI/kaolinite from 2 to 10 GHz. In addition, good microwave absorption performance also relate to magnetic loss. The greater the magnetic loss, the better the microwave absorption performance. The magnetic loss (tan δμ = μ″/μ′) of nZVI/sepiolite was higher than that of nZVI/kaolinite from 2 to 10 GHz (Figure 5D). Therefore, nZVI/sepiolite had higher microwave absorption properties than those of nZVI/kaolinite.

Table 1 presented the chemical compositions of different materials. Kaolinite and sepiolite showed a slight difference in iron content. However, there was a significant difference in iron content between nZVI/kaolinite and nZVI/sepiolite, indicating different loadings of iron.

**Table 1:** Chemical composition of kaolinite, nZVI/kaolinite, sepiolite, and nZVI/sepiolite.

	**Si**	**Al**	**Fe**	**K**	**Ti**	**Na**	**Ca**	**Mg**
Kaolinite	21.65	18.93	0.347	0.637	0.323			
nZVI/Kaolinite	18.60	16.87	9.02	0.518	0.268			
Sepiolite	31.70	3.65	0.676	3.87		6.56	0.669	0.175
nZVI/Sepiolite	27.58	3.03	11.18		0.06	1.00	0.76	2.43

## Degradation of Rh 6G using nZVI/kaolinite and nZVI/sepiolite

nZVI can effectively degrade organic dyes through the following Fenton-like reactions. Fe^0^ was gradually oxidized to Fe^3+^ and generated hydroxyl radicals (HO^•^) and superoxide radicals (${\text{O}}_{2}^{\cdot -}$), which were the main active ingredients for the degradation of Rh 6G.

(1) $$\begin{array}{l} O_{2}+Fe^{0}+2H^{+}\to {\text{Fe}}^{2+}+{\text{H}}_{2}O_{2} \end{array}$$

(2) $$\begin{array}{l} Fe^{2+}+H_{2}O_{2}\to Fe^{3+}+HO\cdot +OH^{-} \end{array}$$

(3) $$\begin{array}{l} Fe^{2+}+O_{2}\to O_{2}^{\cdot -}+Fe^{3+} \end{array}$$

**Figure 6:**
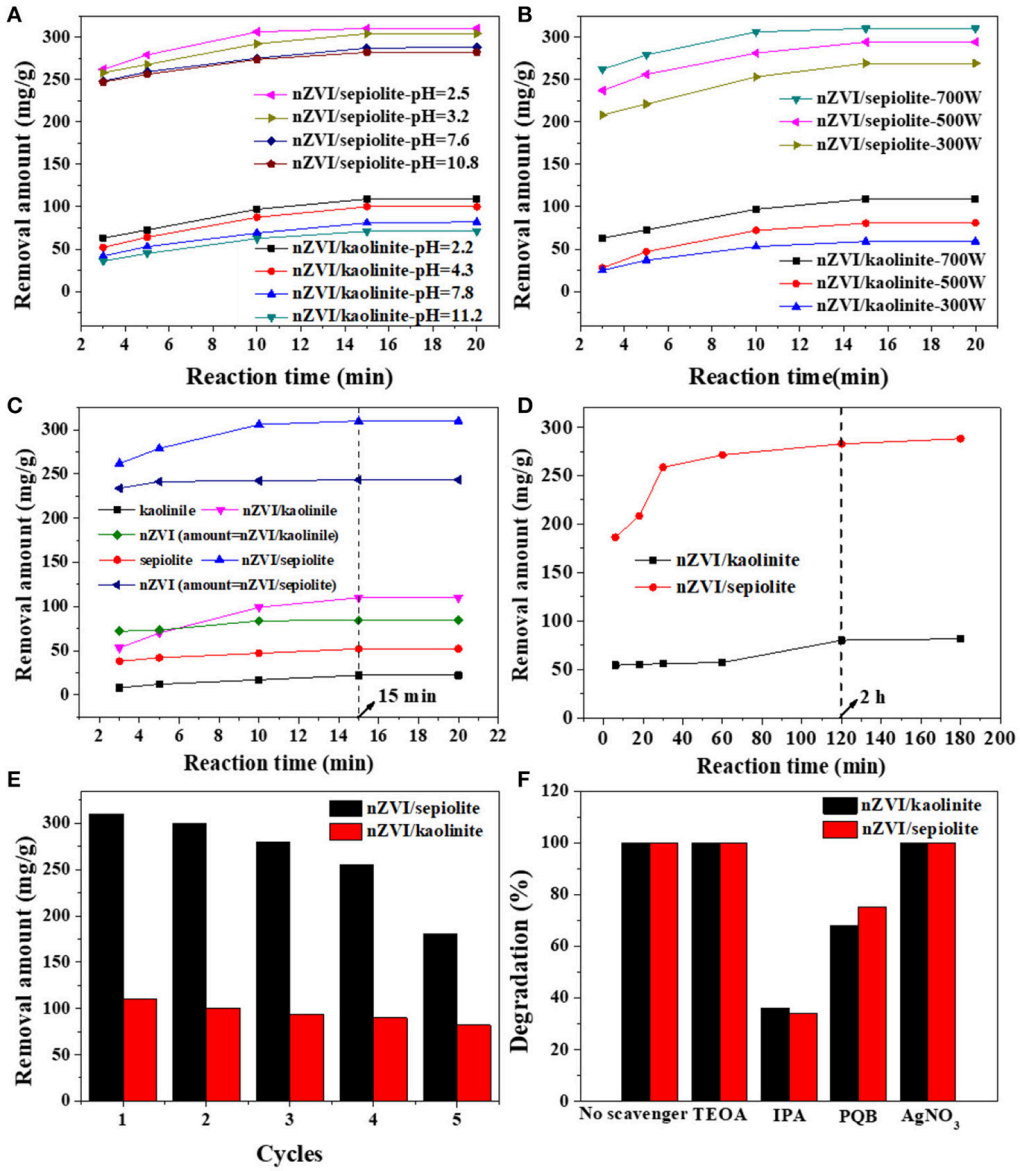
Removal amount Rh 6G with different solution pH **(A)**, microwave power **(B)**, in the presence of different materials under microwave irradiation **(C)**, nZVI/sepiolite, and nZVI/kaolinite without microwave irradiation **(D)**. Cycle stability test for nZVI/kaolinite and nZVI/sepiolite **(E)**. Degradation percentages of Rh 6G with different additives using nZVI/kaolinite, and nZVI/sepiolite under microwave irradiation **(F)**.

The degradation ability of Rh 6G was reflected by the removal amount of Rh 6G under microwave irradiation by nZVI/kaolinite and nZVI/sepiolite. The effect of pH on the degradation rate of Rh 6G was shown in Figure 6A. Both nZVI/kaolinite and nZVI/sepiolite showed higher degradation ability under acidic conditions, indicating that acidic conditions are more suitable. It was because that, under alkaline conditions, the generation of HO^•^ and ${\text{O}}_{2}^{\cdot -}$ would be inhibited and Fe^0^ would react with OH^−^ to form iron hydroxide. Meanwhile, H_2_O_2_ would decompose rapidly when pH > 7. The effect of microwave power on the degradation of Rh 6G by nZVI/kaolinite and nZVI/sepiolite was also investigated (Figure 6B). The removal amount of Rh 6G by nZVI/kaolinite and nZVI/sepiolite increases with the increase of microwave power, since the generation rate of HO^•^ and ${\text{O}}_{2}^{\cdot -}$ are proportional to the microwave power.

Figure 6C showed the degradation abilities of kaolinite, sepiolite, nZVI/kaolinite, nZVI/sepiolite and nZVI (same amount with nZVI/kaolinite or nZVI/sepiolite). The removal amounts of Rh 6G by nZVI/sepiolite, nZVI/kaolinite, sepiolite and kaolinite in 15 min were 300, 110, 52, and 22 mg/g, respectively. The removal amount of Rh 6G by nZVI/sepiolite was better than that of sepiolite and kaolinite adsorption and nZVI/kaolinite degradation. It was possibly caused by the iron content of the materials. We prepared the same amount of nZVI as nZVI/sepiolite and nZVI/kaolinite, whose removal amount of Rh 6G are 243 and 84 mg/g, respectively, indicating that loading nZVI on clay can increase the removal amount of Rh 6G. The higher the negative charge of the layer, the more favorable to adsorb cations. The zeta potentials of sepiolite and kaolinite are −17.4 mV and −11.2 mV, respectively. Therefore, more Fe^3+^ could be adsorbed onto sepiolite and be reduced to Fe^0^, which could generate more free radicals to degrade Rh 6G. In addition, the specific surface area of nZVI/sepiolite is higher than that of nZVI/kaolinite, which could provide more reactive sites for the reaction. What's more, compared with nZVI/kaolinite, the microwave absorption properties of nZVI/sepiolite were also stronger. Without microwave irradiation, the removal amounts of Rh 6G by nZVI/sepiolite and nZVI/kaolinite were 288 and 81 mg/g (Figure 6D), respectively. Although the removal amount of Rh 6G was not significantly improved in the presence of microwave, the equilibrium time decreased from 2 h (Figure 6D) to 15 min (Figure 6C) with the irradiation of the microwave, indicating the presence of microwave could greatly accelerate the reaction. Therefore, microwave is necessary in the process.

NaBH_4_ was used to reduce the Fe^3+^ loaded on the used material to Fe^0^ for the recycle of the composites to investigate the recycling performance of the materials (Figure 6E). There was no significant change in the removal of Rh 6G after three cycles, suggesting that the composites could be reused for several times. We performed an inductively coupled plasma spectroscopy test to check iron leaching to the solution after each reaction, no iron leaching was found, indicating iron has good load stability on clay.

In order to prove the main active ingredient that degrades Rh 6G are HO^•^ and ${\text{O}}_{2}^{\cdot -}$, the same molar concentrations of indolepropionic acid (IPA), p-benzoquinone (PBQ), triethanolamine (TEOA) and silver nitrate (AgNO_3_) were added to solutions during the reaction (Figure 6F). It is well-known that IPA is a typical hydroxyl radical inhibitor and PBQ is a typical superoxide radical inhibitor. The removal amount of Rh 6G dramatically dropped with the addition of these two inhibitors, which indicated that HO^•^ and ${\text{O}}_{2}^{\cdot -}$ were the main active ingredients for the degradation of Rh 6G by nZVI/clay composites.

## Conclusion

In summary, the aggregation and oxidation of nZVI can be effectively inhibited by loading nZVI on clay minerals. When the composites were used to degrade Rh 6G under microwave irradiation, nZVI/sepiolite exhibited better removal performance than nZVI/kaolinite, due to the difference in iron loading amount, which was caused by the difference in specific surface area and zeta potential. In addition, composites could be reused for several times after regeneration. With the addition of different types of scavengers, it was demonstrated that HO^•^ and ${\text{O}}_{2}^{\cdot -}$ were the main active ingredients in the degradation of Rh 6G. It will be a very promising method for future use of nZVI by loading nZVI on clay minerals.

## Author contributions

WR and GL conceived the project. GL and LL designed and performed the experiments. DW analyzed the data. WR and GL wrote the manuscript.

### Conflict of interest statement

The authors declare that the research was conducted in the absence of any commercial or financial relationships that could be construed as a potential conflict of interest.
